# Monoterpene Variation Mediated Attack Preference Evolution of the Bark Beetle *Dendroctonus valens*

**DOI:** 10.1371/journal.pone.0022005

**Published:** 2011-07-19

**Authors:** Zhudong Liu, Bo Wang, Bingbing Xu, Jianghua Sun

**Affiliations:** The State Key Laboratory of Integrated Management of Pest Insects and Rodents, Institute of Zoology, Chinese Academy of Sciences. Beijing, China; Martin-Luther-Universität Halle, Germany

## Abstract

Several studies suggest that some bark beetle like to attack large trees. The invasive red turpentine beetle (RTB), Dendroctonus valens LeConte, one of the most destructive forest pests in China, is known to exhibit this behavior. Our previous study demonstrated that RTBs preferred to attack large-diameter trees (diameter at breast height, DBH ≥30 cm) over small-diameter trees (DBH ≤10 cm) in the field. In the current study, we studied the attacking behavior and the underlying mechanisms in the laboratory. Behavioral assays showed that RTBs preferred the bark of large-DBH trees and had a higher attack rate on the bolts of these trees. Y-tube assays showed that RTBs preferred the volatiles released by large-DBH trees to those released by small-DBH trees. Subsequent analysis revealed that both large- and small-DBH trees had the same composition of monoterpenes, but the concentration of each component differed; thus it appeared that the concentrations acted as cues for RTBs to locate the right-sized host which was confirmed by further behavioral assays. Moreover, large-DBH pine trees provided more spacious habitat and contained more nutrients, such as nitrogen, than did small-DBH pine trees, which benefited RTBs' fecundity and larval development. RTBs seem to have evolved mechanisms to locate those large hosts that will allow them to maximize their fitness. Monoterpene variation mediated attack preference implies the potential for the management of RTB.

## Introduction

Most scolytid bark beetles have specialized feeding habits, and attack one or a few closely related host-tree species [1], [2]. This probably resulted from adaptive radiation when colonizing many niches provided in part by a variety of trees secondary chemicals [3], [4], and from a long evolutionary association of bark beetles with their host trees [5]. Host selection in phytophagous insects consists of a sequence of behavioral responses to an array of stimuli associated with host and non-host plants [6]. The insects in turn are equipped with an array of sensory receptors for visual, mechanical, gustatory and olfactory stimuli [7]. Olfactory cues provided by plants play an important and often interactive role with other stimuli in the host selection process by adults of many species of phytophagous insects [4], [8], [9].

Suitable hosts of bark beetles are distributed unevenly in space and time throughout mixed species forests [10]. Therefore, bark beetles must detect and locate the right habitat, correct host species, and the most susceptible trees within a forest [3], [11], [12]. There is conflicting evidence as to whether bark beetles land on potential hosts at random, making a decision about host suitability at close range (random landing), or whether they are oriented toward host volatiles (primary attraction) [3], [12], [13]–[19]. It is commonly accepted that, after pioneer beetles have initiated attack, the majority of the population orientes to the host in response to secondary attraction [3], [20]. When searching for suitable hosts, especially in mixed forests, flying conifer-inhabiting bark beetles will encounter not only suitable host trees and their odors, but also unsuitable host and nonhost trees. Rejection of these trees could be based on an imbalance of certain host characteristics and/or a negative response to some nonhost stimuli [21], [22].

Some species of scolytidae show a decided preference for colonizing large-DBH trees [23]–[27], which indicates a relationship between bark beetles and host size. How do beetles select and locate large trees? Are they relying on the volatiles released by the host? *Dendroctonus valens*, an invasive bark beetle in China that is native to North America, is known to use host odors or kairomones to locate and select its preferred host, *Pinus ponderosa*, in its native ranges [28]–[31]. Where it is most invasive, in northern China, Sun et al. found that certain ratios of host monoterpenes (α-pinene:β-pinene:3-carene  = 1∶1∶1) and even 3-carene alone efficiently attracted RTBs[32]. Moreover, another study showed the number of *D. valens* attracted to kairomone-baited traps was reduced by nonhost volatiles(NHVs) by 26.3 to 70% [33], a finding confirmed by other studies [34], [35], indicating that NHVs might help beetles to discriminate among potential hosts in the field. This research suggested that primary attraction has an important role in host selection of *D. valens*. Our previous field observations revealed that the beetle primarily attacks large Chinese pine, *Pinus tabulaeformis*, and the previous study suggests that host volatiles may play the crucial identifying role [27]. However, the chemical mechanism underlying such a preference and its adaptive significance are unknown.

It is important to understand the behavior of this beetle since *D. valens*, a secondary forest pest in North America, is a destructive invasive pest in China; however, knowledge of its biology and ecology is limited [32], [33], [36]–[43]. Moreover, since this beetle was introduced into Shanxi Province in China in the early 1980s, when unprocessed logs were imported from the west coast of the United States [44], it has spread rapidly to three other adjacent provinces (Hebei, Henan, Shaanxi and Inner Mongolia), and even Beijing, where it has caused the demise of over 6 million healthy *P. tabulaeformis* pines [45]. Since *P*. *tabulaeformis* is extremely important for reforestation in northern China and is widely planted across a large portion of the country, the majority of Chinese pines are probably at risk.

This study, which is based on the phenomenon that the beetle prefers to attack large-DBH trees, addresses the following questions: (1) Do RTB's use kairomones to discriminate between large and small host trees? (2) Do the beetles benefit by attacking large trees?

## Results

In the laboratory, the choice assay showed RTBs preferred the bark with phloem of large-diameter trees (DBH = 30 cm) over that of small-diameter trees (DBH = 10 cm) (Chi-square  = 10.4, *p* = 0.001); furthermore the beetles by no-choice assay had a higher attack rate on the bolts of large-DBH trees than on those of small-DBH trees (Chi-square  = 19.742, *p*<0.0001). When RTBs were exposed to volatiles in the olfactory test, both female and male adults were shown to be more sensitive to volatiles from the bark of the large-DBH tree than to the bark of the small-DBH tree (female: Chi-square  = 5.488, *p* = 0.019; male: Chi-square  = 10.756, *p* = 0.001) (Table 1).

**Table 1:** Analysis of RTB attacking behavior on large- and small-diameter Chinese pine *P. tabulaeformis.*

Sex		N	DBH 30 cm	DBH 10 cm	Chi-square	*p*
Attacking choice		67	37	14	10.4	0.001^**^
Attacking rate			21 (54)	6 (8)	19.742	<0.0001^*^
Y-tube	Female	41	28	13	5.488	0.019^*^
	Male	41	31	10	10.756	0.001^**^

Values in parentheses are numbers of sample size.

(*) means p<0.05; (**) means p<0.01; (***) means p<0.0001.

**Figure 1:**
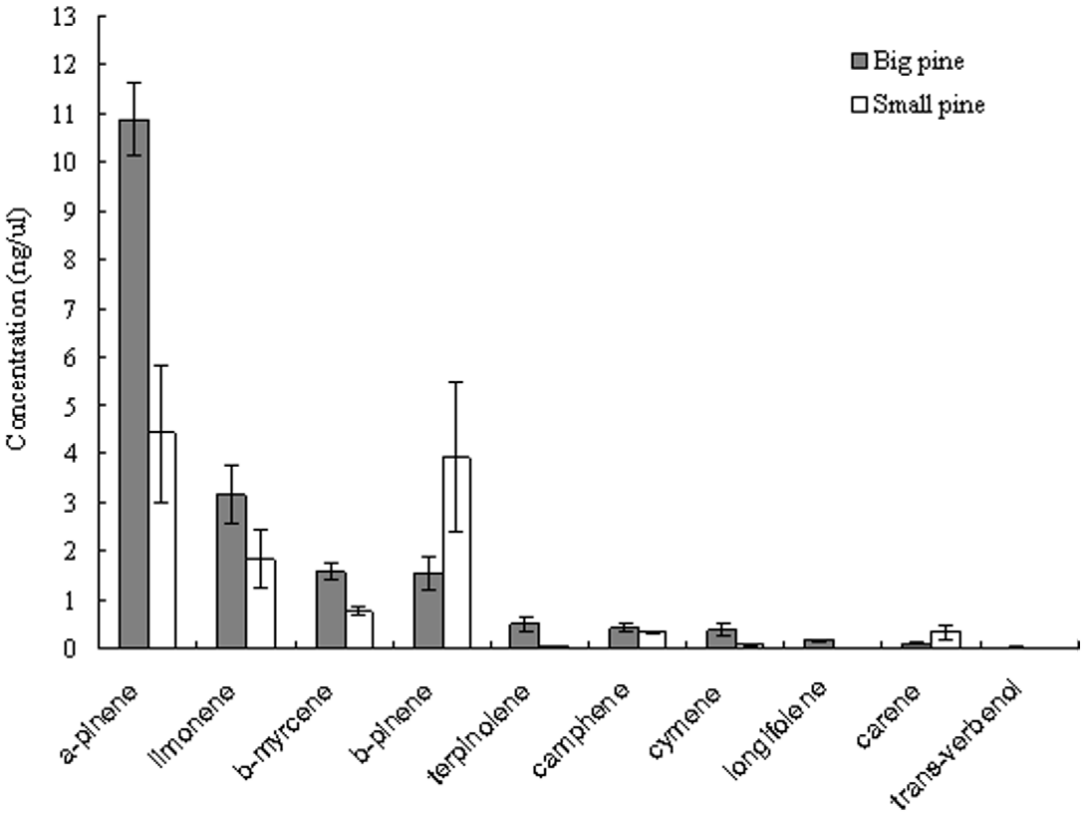
Quantitative variation in monoterpenes of large and small trees of Chinese pine *P. tabulaeformis*.

Analysis of volatiles by GC-MS showed that for both sizes of host trees, the components of the bark did not differ qualitatively according to the composition of monoterpenes (Fig. 1). There were, however, significant differences in the quantitative monoterpene profiles of the different tree sizes. α-pinene, β-pinene, β-myrcene and limonene were main components of volatiles for both large and small-DBH pine trees. Blends A and B, mixed with main components by their estimated natural proportions, elicited significantly different responses from the beetles (Table 2): blend A (produced by large trees) was more attractive than blend B (produced by small trees).

**Table 2:** Behavior assay of RTB with synthetic blend by monoterpene profile of large- and small-diameter Chinese pine *P. tabulaeformis.*

	Sex	N	Blend A	Blend B	Chi-square	*p*
Y-tube	Female	35	26	9	8.257	0.004^**^
	Male	30	26	4	16.137	<0.0001^***^

(*) means p<0.05; (**) means p<0.01; (***) means p<0.0001.

**Figure 2:**
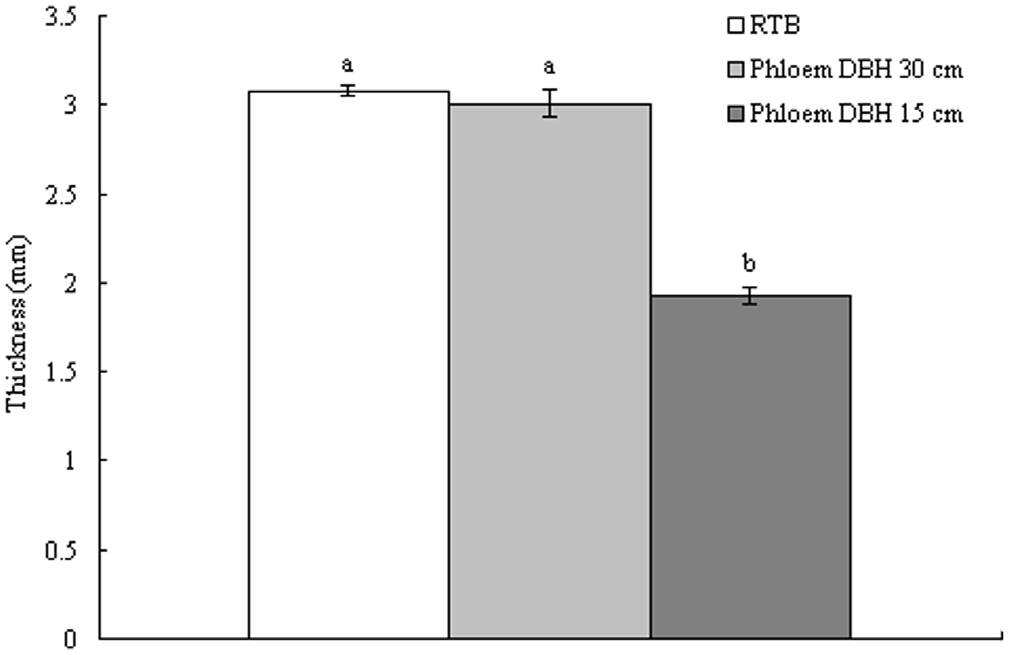
The comparison of RTB body thickness and two size host-phloem thickness. Bars indicate standard errors and different letters on bar indicate significant differences at p≤0.05 with Bonferroni Multiple Comparison (ANOVA).

The phloem was thicker on large-DBH trees than on small-DBH trees (F = 518.3; df = 1, 12; *p*<0.0001) (Table 3) , in which the phloem of large tree was nearly the same thickness as RTB beetles themselves and both were significantly thicker than the phloem of small tree (F = 63.524; df = 2, 103; *p*<0.0001) (Fig. 2). The water content of phloem from large-DBH trees was much higher than that from small-DBH trees (F = 32.133; df = 1.4; *p* = 0.005). More important, the nitrogen content of the phloem, the main limiting source for herbivores, was also much higher in large-DBH trees than in small-DBH trees (F = 31.282; df = 1.4; *p*<0.001), although the amount of soluble sugar in phloem did not differ significantly (F = 2.176; df = 1.4; *p* = 0.162) (Table 3).

**Table 3:** Comparison of pine phloem characteristics between large- and small-diameter Chinese pine *P. tabulaeformis.*

	Phloem thickness (mm)	Water content (%)	Nitrogen content (mg/100 mgDW)	Sugar content (mg/mgDW)
DBH 10 cm	1.93±0.03	59.89±0.56	0.440±0.003	0.119±0.006
DBH 30 cm	3.02±1.46	65.63±0.84	0.633±0.037	0.108±0.003
df	1, 12	1, 4	1, 4	1, 4
F	518.3	32.133	31.282	2.176
*p*	<0.0001	0.005	<0.0001	0.162

The data are shown as mean ± SE.

The fitness experiment showed 17 and 15 of 28 pairs bored into the bolts of large- and small-DBH pine trees, respectively (Table 4: Chi-square  = 0.292, *p* = 0.589); however, among these pairs, 16 had offspring in large-DBH trees and only 7 had offspring in small-DBH trees (Table 4: Chi-square  = 8.876, *p* = 0.003). Furthermore, among those that had offspring, 16 showed neonates in large-DBH trees and only 4 showed neonates in small-DBH trees (Table 4: Chi-square  = 15.462, *p*<0.0001). Moreover, RTBs inoculated into bolts of large-DBH trees had higher fecundity than those inoculated into small-DBH pine trees (Table 4: t = 2.954, *p* = 0.018) and their larvae were significantly heavier on large-DBH compared to small-DBH trees (Table 4: F = 51.128, *p*<0.0001).

**Table 4:** Fitness analysis of RTBs introduced to different size Chinese pine P. tabulaeformis

	No. tested pairs	No. bored in	No. produced offspring	No. had larvae	Fecundity^a^	Weight of larvae (mg)^b^
DBH 30 cm	28	17	16	16	80.9±17.0	4.4±0.2
DBH 10 cm	28	15	7	4	27.4±12.5	2.3±0.2
Chi-square		0.292	8.876	15.462	2.954	51.128
df		1	1	1	8	1, 201
p		0.589	0.003^**^	<0.0001^****^	0.018	<0.0001^****^

Data are shown as Mean ± SE.

a Data were analyzed with paired t-tests.

b Data were analyzed with ANOVA.

## Discussion

Primary attraction and random landing are the two main hypotheses about how beetles locate and select hosts [15], [16], [46]. Burnell suggests that beetles attack trees randomly and that large trees are killed most often because they present beetles with the largest landing targets [47]. Others claim bark beetles used green leaf volatiles to locate host habitats from far away and then find the right host individuals through host-released volatiles [12]. The behavior of RTBs is thought to reflect to the latter hypothesis since *D. valens* is known to use host odors or kairomones to locate and select its preferred host in both native ranges [28]–[31] and in areas where new invasions are occurring, northern China [32], [48]. Our current work strongly suggests that the choice RTBs make between large- and small-DBH host pine trees is governed at least in part by the monoterpene variation in the composition of the trees' volatile compounds; however, we did not test other possible cues such as the visual cues mentioned by Campbell and Borden [49], [50]. The ratio of monoterpenes changes in foliage volatiles when a host becomes old [51], [52] or in bole of sympatric species of conifers [53]. Although monoterpene compositions was qualitatively similar between large- and small-DBH trees, our research shows that quantitative variation in monoterpene composition may facilitate beetles' ability to discriminate between hosts, as hypothesized by Pureswaran et al. [53] and Tomlin et al. [54]. In our study, the changed ratio of monoterpenes due to host size difference explained beetles' attack behavior, which was the first time *D. valens* had been shown to use the monoterpene variation between host sizes to locate the large pine trees.

Other scolytid beetles have also shown a decided preference for colonizing large-DBH trees [23], [24], [26]; such a preference is sometimes attributed to host susceptibility. It has been reported that phloem thickness increases as diameter increases, and that phloem thickness is related to pine vigor [55], [56]. In general, the bigger the DBH, the older the tree, the weaker its vigor [24]. In our study, a pine with DBH of 30 cm is about 60 years old (communication with local forest workers), and this is the size RTBs prefer to colonize. Moreover, our data showed large-DBH trees contained more water than small trees, which supports the view that there is a relationship between a tree's susceptibility to RTB attack and its water content [23], [57]–[60]. The hypothesis of growing space based on stand density, i.-e. the density of the stand of trees, provides a mechanism to illustrate how changes in host vigor influence the susceptibility of individual trees to bark beetle attack [26] and may explain why RTBs prefer to attack large-DBH trees. In this study, however, we did not measure the vigor of pine trees or evaluate how the RTBs determine host susceptibility. Future studies may investigate the relationship between tree vigor, monoterpene variation, and host susceptibility.

Why have RTBs evolved a preference for large-diameter trees? Besides the water content variation mentioned above, large-DBH trees have thicker phloem and contain more nutritional components, such as nitrogen, which may benefit RTBs. *Dendroctonus* adults are known to burrow through the outer bark to the inner bark (phloem) to lay their eggs, larvae then feed on phloem until they pupate, large-DBH trees with thick phloem could provide many advantages since the thickness of phloem have been suggested as potential limits to bark beetle colonization success [58]–[61]. These large trees have thicker phloem than do their small counterparts, which may provide adults with the space to make tunnels and the material for their larvae to feed on. Moreover, the thickness of the phloem of large-DBH trees is nearly the same as the thickness (dorsal-ventral) of the beetle; in contrast, the thickness of the phloem of small trees is less than the thickness of the RTB (Fig. 2). It is likely easier for RTBs to make tunnels in thick phloem because they do not need to spend as much energy to remove the outer bark and encounter fewer obstacles passing through the tunnels.Furthermore, fitness assay did show that large-DBH trees can support more offspring per unit of surface than small-DBH trees can, which ahs been confirmed by Klein et al. [62].

In addition, the thicker phloem of the large-DBH pine trees contains more nutrition, especially nitrogen, which would be a selective factor for RTBs. Nitrogen is a limited resource for many organisms since the nitrogen content of plant tissue is very low relative to that of herbivores, particularly for bark beetle [63]. As a consequence, dietary nitrogen can limit the growth and reproduction of herbivores, and insects will select for attributes that increase nitrogen acquisition [64]. In particular, *Dendroctonus* bark beetles that feed on pine cambium and phloem, a nitrogen-poor substrate, face the challenge of a nitrogen deficit. The nitrogen content of phloem in large-DBH trees was much higher than that of small-DBH trees. The fitness experiment showed RTBs inoculated into the large-DBH trees had more offspring and their offspring developed faster, indicating the respective contribution to RTB development made by thick and thin phloem. Moreover, beetle size is also reported to increase as the amount of nitrogen in the phloem increases [65]. The thick phloem of large-DBH trees contains a large amount of nitrogen, which is a stable index for selection. RTBs prefer to attack large-DBH trees and the preference of RTBs for large-DBH trees is a long-term adaptation to improve fitness.

RTBs are still a major forest pest in China and they seem to be spreading: Inner Mongolia, Henan, Hebei, Shanaxi, and even Beijing have reported infestations. Field investigations indicate that large-DBH trees can support up to about 2000 larvae/tree when they are colonized by RTBs (our unpublished data). Thus it is critical to prevent these trees from becoming infested. Our results can help RTB management become more efficient. Using new techniques such as attractants and repellents as well as nonhost volatiles to manage RTBs might protect large pine trees from attack, which would make substantial contribution to RTB management in future.

## Materials and Methods

### Insects

Lindgren funnel traps with kairomone lure (α-pinene:β-pinene:3-carene  = 1∶1∶1) were used to catch adult RTBs at their over-wintering sites from early May to early June 2010 when the peak of emergence occurs. Field trapping was conducted in a natural distribution of Chinese pine *P. tabulaeformis* at Beishe Mountain at the foot of the Luliang Mountains (N 37° 48′, E 111° 57′, average elevation 1400 m), west of the city of Gujiao, Shanxi Province. This site, dominated by *P. tabuliformis* plantations that are some 30 years old, is where the first outbreak occurred in 1999. Traps were checked every other day and adult RTBs were collected. RTBs were sexed according to the sound of stridulation released by males [66] and the body thickness of about 100 RTBs of each sex was measured (dorsal-ventral) using a micrometer (accuracy 0.02 mm). After that, adult RTBs provided with a phloem-powder-based artificial diet were taken to the laboratory and kept in a climate chamber at 25°C and 55% RH under a photoperiod of L:D 14∶10 for following experiments.

### Attacking behavior of RTBs in the laboratory

Two diameter trees, i–e., small pine trees with DBH of 10 cm and large trees with DBH of 30 cm, were selected in the natural distribution described above. Five trees of each size were randomly selected in the valley, cut into bolts from the bottom section of each tree (ca. 100 cm long each and 2 bolts from each tree) and transported to the laboratory. Bolts were placed upright at 20°C in a temperature-controlled room with natural light coming through the window, and their cut ends were coated with melted wax to retard moisture loss. Two experiments were carried out: one tested whether RTBs preferred to attack the bark of large- or small- DBH trees (dual-choice experiment), the other analyzed the rates of attack on bolts (no-choice experiment).

Bark including outer and inner from both large- and small-DBH bolts was stripped and cut to the same thickness. Two rectangular-shaped pieces of bark (10 cm×5 cm×0.5 cm), one from the bolt of a large-DBH tree and the other from the bolt of a small-DBH tree, were placed between double glass plates covered with preservative film to prevent RTBs from escaping. One pair of RTBs, a female and a male, were released into the space between the glass plates. Starting 24 hours later and continuing for 3 days, plates were checked to observe which piece of bark was bored into successfully by the RTBs. 67 replicates were carried out.

To measure the attack rate on bolts, pairs of adult RTBs (one female and one male) were introduced simultaneously into the bolts of large- and small-DBH pine trees; more than 50 replicates for each treatment were carried out. Each paired set of beetles was enclosed within a transparent 10 ml plastic centrifuge tube, and tubes were fixed on the trunk surface with fibrous lines, preventing the beetles' escape and enabling observation. The beetles were checked daily for a week and successful attacks were counted; success was defined by RTBs boring into the bark. The attack rate was calculated by dividing the number of successful attacks by the total number of tested samples.

### Analysis of volatiles from barks and behavioral assay with Y-tube

Volatiles from bark including outer and inner stripped from both sizes of pine trees were collected with headspace sampling [67]. The bark stripped from each tree was collected in the field at the height of 1 m; 20 cm×24 cm pieces were cut from 8 trees of each size. Bark was enclosed in polyvinyl plastic bags (Reynolds, Richmond, VA, USA) with an activated charcoal filter tube in the inlet to keep in the airflow. Air was drawn by vacuum pump for 5 hr at 150 ml/min through a Teflon tube (3.0 mm ID×50 mm) containing 30 mg Porapak Q, 80/100 mesh (Alltech Associates Inc., IL, USA), with glass wool (silane treated) fixed at both ends. After these volatiles were collected, the Porapak Q traps were rinsed with 1000 µl hexane (Fluka puriss p.a.), and the rinsed solution was kept in Hewlett Packard 2-ml vials. The extracts were filtered through a Pasteur pipette column containing 15 mm Na_2_SO_4_ and a plug of glass wool and stored at -20°C for later GC-MS analysis and behavioral assay.

Five extractions of each group were analyzed on a Hewlett-Packard (HP) 6890 tandem gas chromatograph-mass spectral detector (GC-MS) operating with HP-5MS column (30 m length by 0.25 mm ID by 0.25 µm; J&W Scientific, Folsom, CA, USA). The temperature program was 50°C for 2 minutes, 5°C/min to 200°C, and then 20°C/min to 220°C and held for a final 10 minutes. The flow of helium was 1.0 ml/min. Aliquots of extracts (1 µl) were injected with a split model of 50∶1, at 250°C. The compounds were identified by comparing retention times and mass spectra of synthetic standards. Quantification of the volatiles' components in the extracts was done by relating their peak areas to the internal standard, acetic acid heptyl ester.

The other 3 samples from each group were pooled and used for the behavioral bioassay. Bioassays were conducted in a glass Y-tube olfactometer by the method described by Liu *et al*. [43]. Approximately 30 min before trials were initiated, the adult RTBs were introduced into a separate holding container, so they would not be exposed to tested odors before their release. At the beginning of each trial, an individual beetle was released at the downwind end of the Y tube. Each beetle was given 10 min to respond to the treatment, and a choice for the left or right arm of the olfactometer was noted when the beetle went 5 cm past the Y junction. If it failed to select one volatile, a new one was introduced. The arms of the olfactometer were exchanged after each replicate to prevent position effects. Temperature and humidity in the olfactometer were maintained at 25°C and 70%, respectively. At least 40 females and 40 males were tested.

Using the GC-MS results from the volatiles of large- and small-DBH trees, synthetic blends A and B were prepared; four main monoterpenes, α-pinene, β-pinene, β-myrcene and limonene, were mixed in their estimated natural proportions of large- and small-DBH trees (Blend A, the concentration of α-pinene, β-pinene, β-myrcene and limonene was 10.8, 1.53, 1.57, and 3.15 ng/ul, respectively; Blend B, the concentration of α-pinene, β-pinene, β-myrcene and limonene was 4.42, 3.93, 0.75, and 1.84 ng/ul, respectively). All chemicals were commercially available, obtained from Acros, TCI, Sigma and Fluka, respectively. Synthetic blends A and B were used in behavior assays with a Y-tube as mentioned above to confirm whether blend A from large-DBH trees was more attractive for RTBs than blend B from small-DBH trees. At least 30 replicates were carried out for both female and male RTBs.

### Characteristics of phloem

In the field, seven large-DBH trees and seven small-DBH trees were randomly sampled and their bark was stripped at a height of 1 meter. Bark was taken back to the laboratory where an analysis of phloem thickness, phloem nitrogen and nonstructural sugar content was carried out.

The thickness of phloem was measured with a micrometer (accuracy 0.02 mm) and one piece per tree was measured 10 times at different sites; each piece represented one replicate (7 replicates for each).

Six pieces of phloem, one piece from 3 trees/size class, were cut into small pieces and dried for 3 d to a constant mass at 60°C in an oven chamber. The dried pieces were then ground into powder with a heavy duty blender (Waring Commercial, Kent City, MI, USA) and were kept at 4°C until nitrogen and sugar content could be analyzed.

Three subsamples (0.5 g) of each powder of phloem were measured for total nitrogen content with the micro-Kjeldahl procedure (sulfuric acid digestion followed by analysis with a Technicon Auto-Analyzer; as shown by Ayres *et al*. [65]). Nitrogen data were expressed as gram per 100 gram dry phloem.

Hot ethanol was used to extract soluble sugar [68]. For each sample, three subsamples (50 mg) were extracted three times with 5 ml of 80% ethanol by boiling the samples in glass tubes capped with glass marbles in a 95°C water bath for 10 min each. After each extraction, the tubes were centrifuged at 2500 rpm for 5 min, and the supernatants of the three extractions were pooled for sugar analysis. The amount of sugar was determined by the phenol and sulfuric acid method [69]. Absorbance was determined at 490 nm on a MDS spectrophotometer VersaMax (Molecular Devices Corp., Sunnyvale, CA, USA). The results were expressed in mg/mg dw using a calibration curve obtained by measuring glucose standards in seven concentrations ranging from 0.01 to 0.1 mg ml^−1^.

### Fitness analysis of RTBs inoculated into bolts of large- and small-DBH pine trees

The aim of this experiment was to see on which size tree RTBs perform best. A pair of RTBs, one female and one male, was inoculated into the hole drilled with a 1.0-cm-diameter cork borer on a bolt (2 holes drilled per bolt and totally 28 replicates for each size). This pre-drilled hole made it easier for RTBs to bore into the bark. One month after RTBs were inoculated into bolts, the number of those that had bored successfully into bolts was recorded, and the tunnels were dissected to check for the presence of eggs/larvae; the total fecundity (total numbers of eggs and larvae) of each pair was recorded. At this time, eggs and small larvae (most of them neonates) were seen. It was not feasible to weigh larvae from two groups, so the body weight from each group was measured one month later.

### Statistical analysis

RTB attack choice, attack rate, data from the Y-tube test, and fitness data on the number who produced offspring and had small larvae were analyzed using a Chi-square test with the null hypothesis of equal expectation [70]. The component concentration of volatiles, tree characteristics (such as phloem thickness, nitrogen and sugar content), and range of body weights between insects that fed on large- and small-DBH pines were analyzed with a one-way ANOVA [70]. Fecundity was analyzed with paired t-tests.
